# The Clinical and Histological Intersection of Cardiac Sarcoidosis and Giant Cell Myocarditis

**DOI:** 10.7759/cureus.59783

**Published:** 2024-05-07

**Authors:** Toishi Sharma, Kramer Wahlberg, Friederike Keating, Ahmed Harhash, Leslie T Cooper

**Affiliations:** 1 Cardiology, University of Vermont, Burlington, USA; 2 Internal Medicine, Mayo Clinic, Jacksonville, USA

**Keywords:** pet scans, biopsy, management, overlap, histology, giant cell myocarditis, cardiac sarcoidosis

## Abstract

The clinical and imaging features of cardiac sarcoidosis (CS) and giant cell myocarditis (GCM) are occasionally indistinguishable. This is a case of heart block and ventricular tachycardia where cardiac MRI, fluorodeoxyglucose positron emission tomography (FDG-PET) and biopsy revealed intermediate clinicohistologic phenotype between CS and GCM. This highlights gaps in the management of overlap conditions.

## Introduction

Cardiac sarcoidosis (CS) and giant cell myocarditis (GCM) differ significantly in their presentation. Cardiac sarcoidosis is an infiltrative cardiomyopathy that results from granulomatous inflammation affecting the heart. One-fourth of patients with sarcoidosis are known to have cardiac involvement. While CS tends to have an insidious onset, GCM usually presents with acute heart failure associated with dangerous ventricular arrhythmias. However, not uncommonly, these entities have a clinical overlap and diagnostic dilemmas may be further confounded by similar imaging and histopathological features [1]. This is a case of heart block and ventricular arrhythmia where cardiac MRI, fluorodeoxyglucose positron emission tomography (FDG-PET) and biopsy revealed intermediate clinicohistologic phenotype between CS and GCM.

## Case presentation

History of presentation

A 51-year-old previously healthy female ski patrol director presented with six weeks of intermittent pre-syncopal episodes. She denied associated chest pain, shortness of breath, cough, palpitations, orthopnea, leg swelling, fever, rash or recent tick bites. Family history was noteworthy for the absence of cardiomyopathy and sudden cardiac death. The patient had no history of recent vaccination, travel to regions endemic for *Trypanosoma cruzi* or known cardiovascular risk factors. On examination, she had intermittent bradycardia with ventricular rates as low as 28 beats per minute (bpm). She felt lightheaded during these episodes that lasted for up to 30 seconds. Her resting heart rate at the time was 72 on heart monitor. Auscultation revealed no murmurs, rubs and gallops. Her jugular venous pressure (JVP) was normal at 7 with absence of cannon A waves in the venous pulse.

Differential diagnosis

Intermittent complete heart block in a young female raised the possibility of potential Lyme’s carditis, infectious or autoimmune disorders including cardiac sarcoidosis, viral myocarditis and electrolyte or endocrine causes like hyperkalemia or thyroid disorder.

Investigations

**Figure 1:**
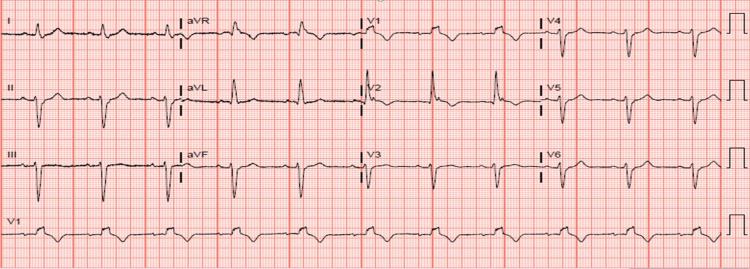
EKG showing sinus rhythm, right bundle branch block and left anterior fascicular block

EKG revealed normal sinus rhythm, right bundle branch block and left anterior fascicular block (Figure 1).

**Figure 2:**
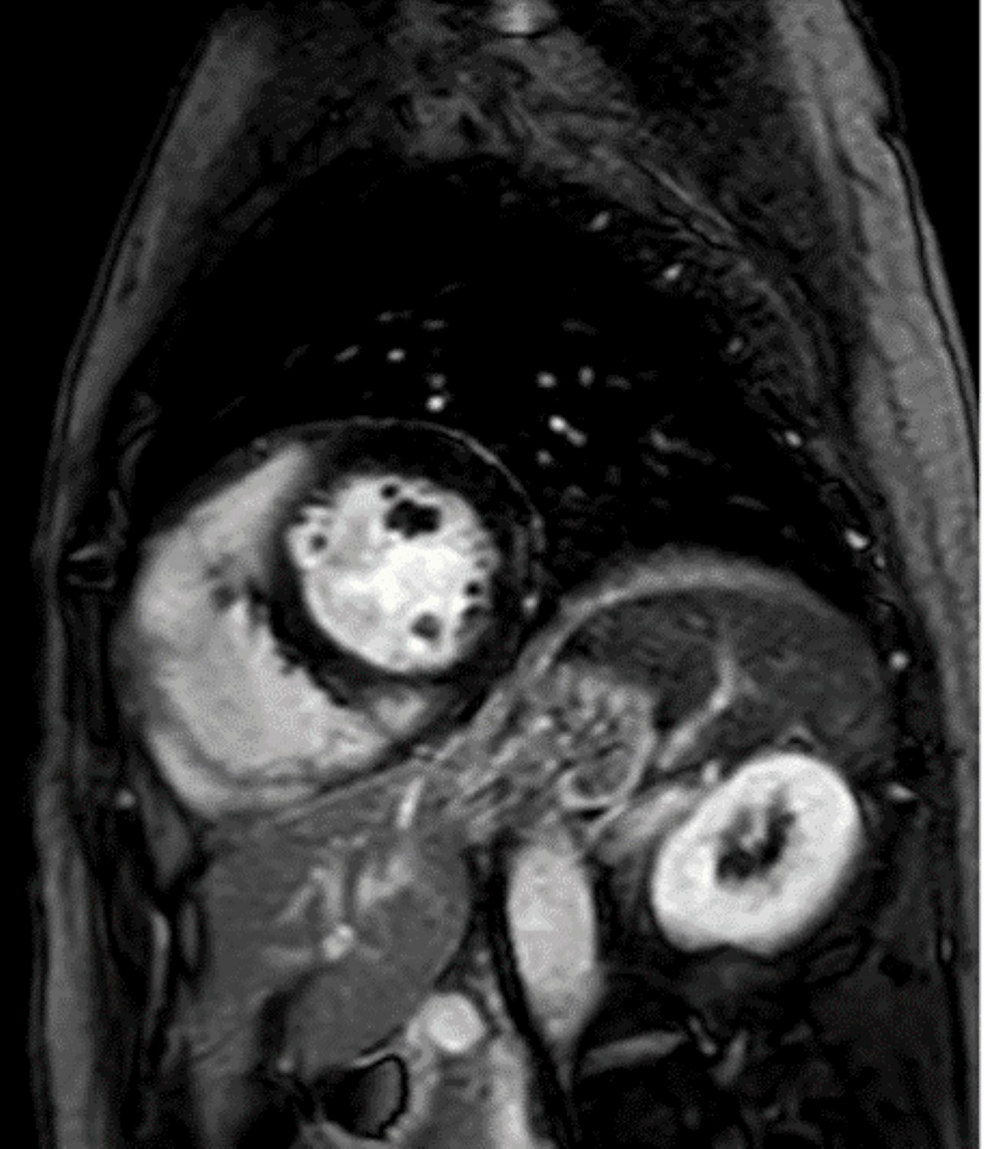
Cardiac MRI (CMR) showing well-demarcated sub-epicardial increased T2 signal suggesting edema in the anteroseptal wall and inferoseptal wall extending from base to apex

Intermittent complete heart block (CHB) was recorded on telemetry. Troponin T, serum electrolytes and Lyme serology were normal. Transthoracic echocardiogram revealed normal left and right ventricular function with normal strain pattern. Cardiac MRI (CMR) demonstrated normal left ventricular size and function with extensive, well-demarcated sub-epicardial increased T2 signal suggesting edema in the anteroseptal wall extending from base to mid-chamber and inferoseptal wall extending from base to apex. T1-weighted myocardial enhancement following gadolinium was present in the same distribution (Figure 2).

**Figure 3:**
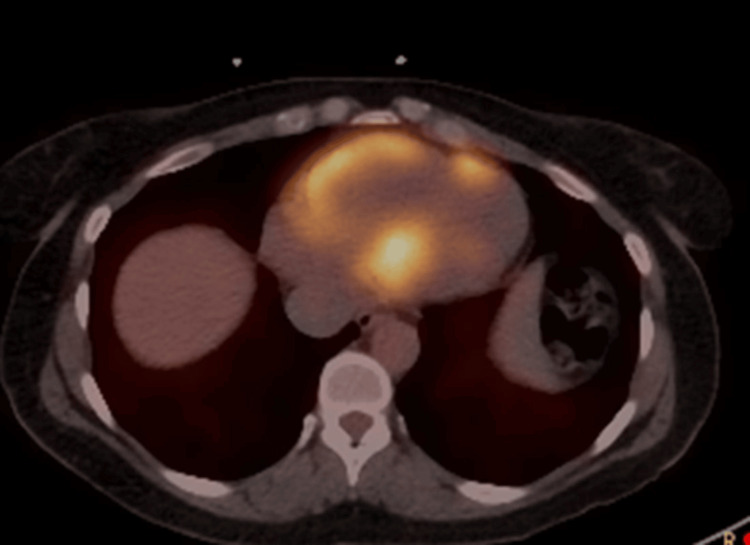
Cardiac PET scan with high right ventricular and septal intake PET: positron emission tomography.

This nonspecific pattern of delayed enhancement combined with elevated T2 values represented active inflammation. Body and cardiac FDG-PET images were remarkable for the absence of any extracardiac focus and the presence of increased metabolism in the right ventricle and septum (Figure 3).

**Figure 4:**
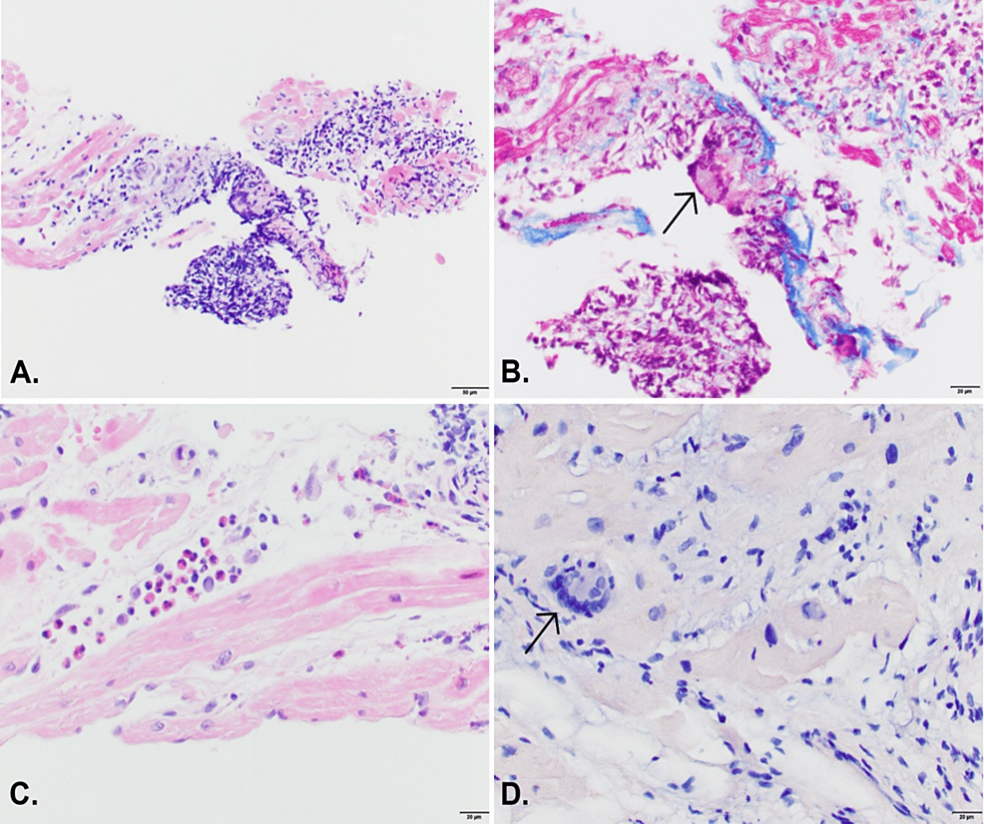
Biopsy photomicrographs showing features of both sarcoidosis and giant cell carditis (A) Medium power view (20x) of H&E-stained section shows a focus of mixed inflammatory cells in the myocardium. (B) Trichrome stain (40x) highlights the early fibrosis associated with the focus of inflammation with a rare giant cell (arrow). (C) High power (40x) of H&E-stained section shows a focus of predominantly eosinophils. (D) Congo red-stained section (40x) is negative for amyloid but shows a giant cell (arrow) without granuloma formation. H&E: hematoxylin and eosin.

The perfusion scans showed no left ventricular perfusion defect. There was absence of abnormal intake or perfusion defect in the region of abnormality seen on CMR. Right ventricular endomyocardial biopsy revealed patchy lymphohistiocytic infiltrate with rare giant cells, scattered eosinophils with focal degeneration of myocytes without definitive granulomas (Figures 4A-4D).

Trichrome satin showed early fibrosis in areas of the infiltrate, while the iron and Congo red stains were negative for hemochromatosis and amyloidosis.

Management 

The patient received a pacemaker for CHB. For possible giant cell myocarditis, she was treated with prednisone and cyclosporine. Two days post discharge, she developed new-onset palpitations and pacemaker interrogation revealed premature ventricular contractions (PVCs). Over the next six weeks, she developed non-sustained ventricular tachycardia (NSVT) and symptomatic sustained VT (rates 140 to 160 bpm) detected on device leading to readmission. Repeat FDG-PET during the admission CMR showed resolution of myocardial abnormalities, i.e., absence of delayed myocardial enhancement and abnormal metabolism. She was initially started on Sotalol 120 mg twice a day (BID) for ventricular tachycardia (VT) suppression; however, due to repeated episodes, Sotalol was held and Amiodarone was started with significant reduction in VT. Her device was upgraded to implantable cardioverter defibrillator (ICD). Her Cyclosporine trough levels were therapeutic at 339 ng/ml during re-admission.

## Discussion

Giant cell myocarditis (GCM) typically has a more acute presentation with more heart failure and arrhythmias than cardiac sarcoidosis (CS). Imaging features including tissue characterization patterns on CMR and regions of inflammation on FGS-PET often overlap [1]. Endomyocardial biopsy is considered the diagnostic gold standard; however, up to 10% of patients may have features typical of both GCM such as eosinophils and poorly formed granuloma more typical of sarcoidosis [2,3]. The diagnosis of CS requires the presence of at least one non-caseating granuloma with or without lymphocytic myocarditis or giant cells, while the diagnosis of GCM requires multinucleated giant cells, active myocyte necrosis and extensive inflammation [4]. Nordenswan et al. found that rather than histopathologic diagnosis, the key determinant of prognosis between GCM and CS appears to be the extent of myocardial injury [5]. Similarly, Gilotra et al. found that the prevalence of “sarcoidosis-related cardiomyopathy” is increasing [6]. Our case fits in the category of CS and GCM clinical and histological overlap. Considering the significant differences in disease course, management and prognosis between GCM and CS with limitations of myocardial biopsy, research using RNA sequencing for analysis of both sarcoid and GCM transcriptome is underway which will help to identify transcriptional heterogeneities and novel therapeutic targets in future [7].

## Conclusions

A substantial minority of cases initially thought to be GCM are found on expert review to be more likely cardiac sarcoidosis. Rare giant cells can be seen in sarcoid, which is also favored by a predominance of fibrosis vs necrosis. However, approximately 10% of cases have true overlap with histological features of both disorders and intermediate risk of death or cardiac transplantation. When cardiac sarcoid is being considered, ICD should be strongly considered even in the absence of ventricular ectopy or reduced ejection fraction. Ventricular arrhythmias may not correlate with disease activity on CMR or FDG-PET.
